# Multiscale Fabrication Process Optimization of DFB Cavities for Organic Laser Diodes

**DOI:** 10.3390/mi15020260

**Published:** 2024-02-10

**Authors:** Amani Ouirimi, Alex Chamberlain Chime, Nixson Loganathan, Mahmoud Chakaroun, Quentin Gaimard, Alexis P. A. Fischer

**Affiliations:** 1 Laboratoire de Physique des Lasers, UMR CNRS 7538, Université Sorbonne Paris Nord, 99 Avenue JB Clément, 93430 Villetaneuse, France; alexchamberlain.chime@univ-paris13.fr (A.C.C.); chakaroun@univ-paris13.fr (M.C.); quentin.gaimard@univ-paris13.fr (Q.G.); fischer@univ-paris13.fr (A.P.A.F.); 2 Centrale de Proximité en Nanotechnologies de Paris Nord, Université Sorbonne Paris Nord, 99 Avenue JB Clément, 93430 Villetaneuse, France; nixson.loganathan@univ-paris13.fr; 3 Institut Universitaire de Technologie FOTSO Victor, Université de Dschang, Bandjoun P.O. Box 134, Cameroon

**Keywords:** OLED, micro-cavity, DFB, e-beam lithography, process optimization, OLD

## Abstract

In the context of the quest for the Organic Laser Diode, we present the multiscale fabrication process optimization of mixed-order distributed-feedback micro-cavities integrated in nanosecond-short electrical pulse-ready organic light-emitting diodes (OLEDs). We combine ultra-short pulsed electrical excitation and laser micro-cavities. This requires the integration of a highly resolved DFB micro-cavity with an OLED stack and with microwave electrodes. In a second challenge, we tune the cavity resonance precisely to the electroluminescence peak of the organic laser gain medium. This requires precise micro-cavity fabrication performed using e-beam lithography to pattern gratings with a precision in the nanometer scale. Optimal DFB micro-cavities are obtained with 300 nm thick hydrogen silsesquioxane negative-tone e-beam resist on 50 nm thin indium tin oxide anode exposed with a charge quantity per area (i.e., dose) of 620 µC/cm^2^, developed over 40 min in tetramethylammonium hydroxide diluted in water. We show that the integration of the DFB micro-cavity does not hinder the pulsed electrical operability of the device, which exhibits a peak current density as high as 14 kA/cm^2^.

## 1. Introduction

Within the realm of organic optoelectronics, various devices, such as organic light-emitting diodes (OLEDs) [1], organic photovoltaics (OPV) [2,3], and organic photodetectors (OPDs) [4,5], have been successfully demonstrated, reaching the market stage. However, a device type that still awaits unambiguous demonstration is the organic laser diode (OLD), which holds the potential to disrupt conventional laser diodes thanks to its simple and low-energy fabrication process and environmental-friendly organic semiconductors. They could pave the way for a credible and innovative low-technology organic photonic platform, with numerous applications in optical communication, Internet of Things, biology, and medical science, among other things [6].

In this direction, numerous studies have been carried out to synthesize new organic materials, allowing for ever higher net laser gains [7,8]. However, a significant hurdle in attaining the laser threshold through electrical excitation is the integration of a high-quality factor micro-cavity in the OLED organic heterostructure, allowing for low laser thresholds. According to the literature, the mixed-order distributed feedback (DFB) cavity is reported to have the lowest laser threshold under optical pumping [9,10,11,12,13,14]. A recent paper, published in 2019 by Adachi’s group, demonstrated promising results using such a cavity [15]. Since then, no other study has been reported, and, in particular, the process of micro-cavity fabrication has not been detailed. This is because the integration of a DFB cavity in an OLED poses several fabrication challenges. One of the first challenges is to combine ultra-short pulsed electrical excitation and the laser cavity. This requires the integration of a highly resolved DFB micro-cavity with an OLED stack and with microwave electrodes. The latter allows for the delivery of nanosecond and sub-nanosecond electrical pulses to an OLED, which is necessary to achieve intense excitation levels in the kA/cm^2^ range so as to reach the laser threshold. A second challenge is to tune the cavity resonance wavelength defined by Bragg’s law precisely to the electroluminescence peak of the organic laser gain medium to maximize gain. This challenge on the precision of the cavity resonance wavelength is, in fact, a double challenge; indeed, according to Bragg’s law, the precision of the DFB cavity resonance wavelength depends on the precision on the DFB period and on the refractive indices of the materials present in the cavity.

The precision on the effective refractive indices was presented in a previous study [16], where the different dimensions of the optimal grating were also calculated and presented. However, the process of fabrication of the DFB cavities was not presented in detail. Moreover, in the previous paper, electrodes were not included, while the latter had an impact on the fabrication process parameters. In [16], different cavities without electrodes were tested under optical pumping to identify, among the different geometries, those allowing laser emission.

In the current study, we focus on the fabrication challenges of a DFB laser cavity compatible with ultra-short pulsed electrical excitation to meet the first challenge. This includes the fabrication of millimetric microwave electrodes before the fabrication of the DFB photonic nanostructure, which leads to a multiscale technological challenge [17]. Indeed, the device consists of an organic heterostructure of millimetric size integrated with a DFB photonic nanostructure, meeting the second challenge, which is implanted with micrometric precision between microwave electrodes. The integration of these three elements requires multiscale solutions, both for patterning and aligning them with respect to each other.

This work details the optimization of this fabrication process while proposing a solution for the multiscale challenge in patterning and alignments. We first specify the cavity geometry before describing the fabrication process. Subsequently, we delve into the optimization of the fabrication process, focusing on key parameters, such as anode thickness and development time as a function of the electron quantity per area unit (dose), while also addressing the encountered challenges. Finally, we showcase the fabricated cavity and its integration on the electrodes.

## 2. Materials and Methods

### 2.1. Cavity Presentation and Design

**Figure 1:**
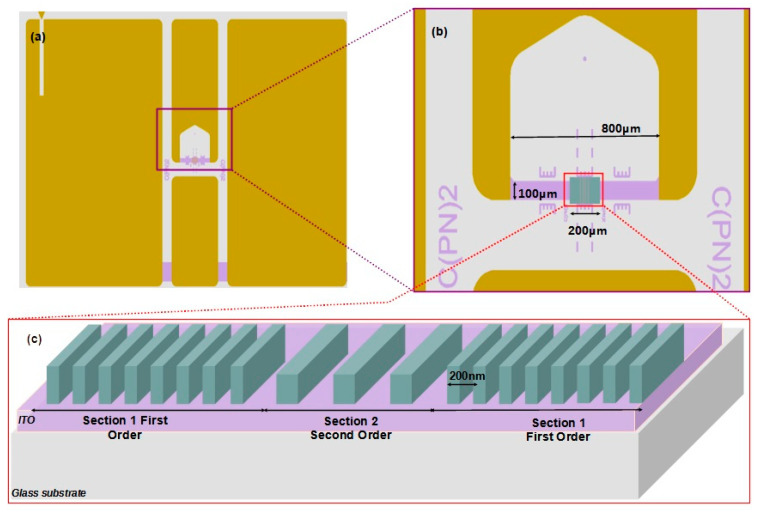
Integration of the DFB micro-cavity on the electrodes: (**a**) top view of the coplanar waveguide electrodes, (**b**) position of the DFB micro-cavity relative to the electrodes, and (**c**) structure of the multi-section mixed-order grating of the DFB micro-cavity.

The micro-cavity is divided into three sections, as illustrated in Figure 1c; Section 1 (on the left) and Section 3 (on the right) consist of first-order gratings. These gratings play the role of mirrors, effectively confining the light within the cavity’s plane. Section 2 is a second-order grating. It serves two purposes: firstly, it acts as spacer between the mirrors defining the free spectral range of the cavity; secondly, it functions as an out-coupler, allowing the light to exit the cavity in a direction perpendicular to its plane [18].

The resonance wavelength of the grating is given by the well-known Bragg’s law:

(1) $$\lambda_{\mathrm{Bragg}}=2\;n\Lambda$$

where Λ is the period of the structure, and n is the effective refractive index. The relative uncertainty on the cavity resonance wavelength is determined by the following expression:

$$\frac{\Delta \lambda_{Bragg}}{\lambda_{Bragg}}=\frac{\Delta n}{n}+\frac{\Delta \Lambda }{\Lambda }$$

To take into account the index difference between the line and the interline spacing, we adapt the Bragg’s law in a combined optical waveguide and quarter-wavelength approach; the period of the structure is made of a line width *Λ_L_* and a spacing between lines *Λ_H_* with an index $n_{L}$ for the lines and an index $n_{H}$ for the interline spacing. The resulting Bragg law follows:

(2) $$\lambda_{\mathrm{Bragg}}=2\left(n_{L}\Lambda_{L}{+n}_{H}\Lambda_{H}\right)$$

The first-order sections consist of the periodical alternation in low-index quarter-wavelength lines of width $\Lambda_{L}=\lambda /4n_{L}$ spaced by a high-index quarter-wavelength layer of width $\Lambda_{H}=\lambda /4n_{H}$. They start and end with high-index layer (interline spacing). As with quarter-wavelength multilayered mirrors, each mirror is made of *N* pairs plus a single high-index layer. In a previous study [16], we identified that *N* = 250 is necessary to maximize the reflectance and the quality factor. The central section (Section 2 Figure 1c) consists of alternating low-index half-wavelength lines of width $2\Lambda_{L}$ and high-index half-wavelength interline spacings $2\Lambda_{H}$. This starts and ends with a low-index layer ($n_{L}$). To limit the free spectral interval of the cavity, Section 2 is made of *M* = 19 pairs plus one single low-index line [16].

Ideally, the targeted Bragg’s wavelength $\lambda_{\mathrm{Bragg}}$ should correspond to the electroluminescence peak of the laser gain material, which for 2-(2-(4-(Dimethylamino)styryl)-6-methyl-4H-pyran-4-ylidene)malononitrile (DCM) is $\lambda_{\mathrm{DCM}}=622\;\mathrm{nm}$ [19]. However, it has been shown that re-absorption by singlet excitons leads to the highest net gain when the cavity is red-shifted from the emission spectrum [20].

With $\lambda_{\mathrm{Bragg}}=622\;\mathrm{nm}$, $n_{L}=1.45$ as refractive index for SiO_2_ (HSQ) lines [21] and $n_{H}=1.7$ as refractive index for organic materials (Alq3) [22]; the width of low-index quarter-wavelength line is $\Lambda_{L}=107\;\mathrm{nm}$ and the high-index quarter-wavelength interline spacing is $\Lambda_{H}=91\;\mathrm{nm}$. With effective instead of bulk refractive indices, values of $\Lambda_{L}$ and $\Lambda_{H}$ are both larger.

In order to obtain a laser resonance above 90% of the maximum of the electroluminescence spectra, the targeted wavelength range is Δλ=37 nm. Assuming a relative uncertainty on the refractive index that is negligible, ΔΛ the relative uncertainty on the grating period must be about 5%. To fulfill this condition, the resolution of both $\Lambda_{L}$ the line width and $\Lambda_{H}$ the interline spacing is about ΔΛ=5 nm. This resolution requires the use of the electron beam lithography technique. Nanometric precision is challenging to achieve, even with e-beam lithography, which is the reason why fine optimization is necessary.

### 2.2. Fabrication Methods

**Figure 2:**
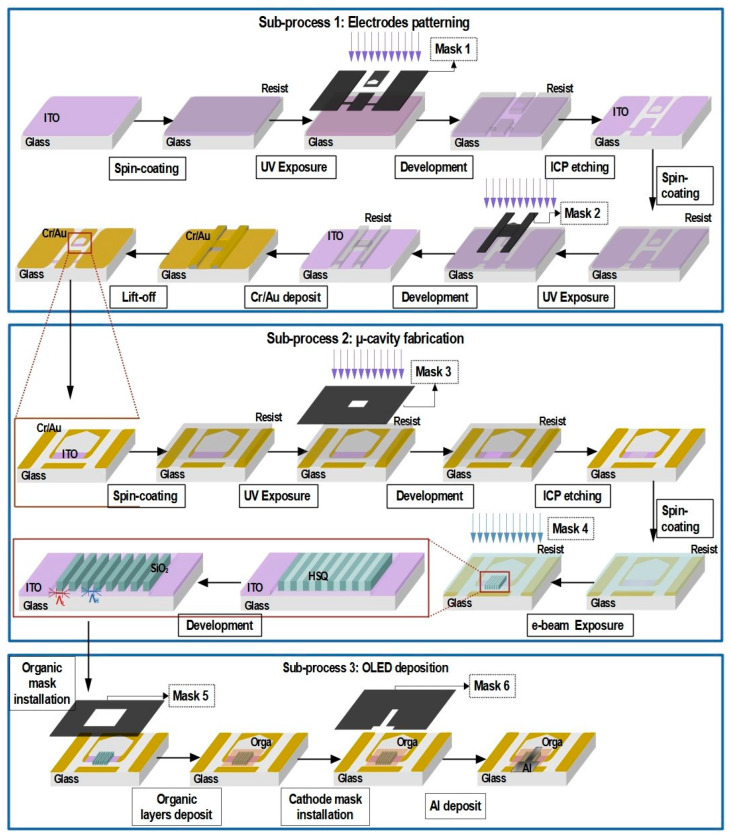
Device fabrication steps: 3 sub-processes and 20 sub-tasks.

The fabrication of the high-speed OLED with integrated mixed-order DFB cavity requires four main sub-processes, which are divided into sub-tasks, as illustrated in Figure 2. In the current work, we further detail, in sub-process 2, the integration of micro-cavity on the anode.

The substrates consist of 700 µm thick glass samples measuring 17 × 25 mm^2^, covered with a transparent and conductive layer made of indium tin oxide (ITO). The thickness of this layer will be discussed below. The first sub-process consists of the electrode patterning and is performed in two main steps: Firstly, the ITO layer is dry etched using the coplanar waveguide (CPW) geometry defined by Mask 1. The geometry dimensions of CPW electrodes were reported in [23]. CPW electrodes ensure impedance matching between the high-speed OLED and the nanosecond electrical pulse driver. Secondly, partial gold coating is performed using the metallization mask (Mask 2). Each step is achieved through a photolithography step, as presented in Figure 2. Intermediate ends of the CPW electrodes are terminated with unmetallized 100 µm large and 800 µm long ITO arms (light-purple area in Figure 1b). The 100 µm dimension is chosen to meet the condition required to confer high-speed properties to the OLED, which entails keeping the OLED size in the range of few hundred micrometers [17].

The main step on which the current study focuses is the integration of the micro-cavity onto the anode active zone according to sub-process 2. This fabrication process described in Figure 2 is performed by e-beam lithography. The accelerated e-beam, with a voltage between 1 kV and 30 kV, allows for a higher resolution than photolithography. To define the optimum micro-cavity fabrication parameters, the main investigated parameters are the ITO thickness, the dose, and the time of development. Since an error of only ΔΛ=5 nm on the grating period can result in a Δλ=37 nm shift in the Bragg’s resonance wavelength, the fabrication of the micro-cavity requires nanometer-scale accuracy. Additionally, micrometric alignment is crucial to center the micro-cavity on the anode active area. Indeed, one of the main challenges of this process is to align the micro-cavity on the 800 µm long and 100 µm large ITO arm of the CPW electrodes previously patterned by optical lithography shown in Figure 1a. The thickness of the ITO arm is obtained through an etching step using a photolithography mask (mask 3) that delineates the area to be etched (200 × 200 µm^2^), as presented in Figure 2. To fabricate the DFB micro-cavity, we use a commercially available solution called Fox16, which is a negative-tone e-beam hydrogen silsesquioxane (HSQ) resist. This solution is known for providing a high resolution (<10 nm), making it one of the top choices in its category. Once exposed to e-beam, this resist undergoes a transformation and becomes similar to SiO_2_. This characteristic enables its compatibility with the organic molecule deposition though thermal evaporation, even at temperatures as high as 200 °C. Importantly, this compatibility is achieved without requiring any additional technological steps [24,25]. Adhesion is very sensitive to the surface condition. To avoid any problems, the sample surface is clean with UV-ozone treatment. The resist is spin coated at 6000 rpm with acceleration of 6000 rpm/s. Then, the resist is annealed at 85 °C for 10 min. The HSQ film thickness is 300 nm, as targeted by the modeling study and simulation [16].

The e-beam is configured with an accelerated voltage of 20 kV, a diaphragm aperture of 15 µm, a working distance varied around 6.5 mm depending on ITO thickness, and a pitch current in the 60–80 pA range. As the relevant dose depends on the ability of the sample to evacuate carrier, its value will be different for each ITO thickness. This aspect will be further addressed below.

The development uses a solution containing tetramethylammonium hydroxide (TMAH) (commercial solution MFCD-26 from Rohm and Haas Electronic Materials LLC, Marlborough, MA, USA). While the latter reacts with the e-beam resist, Si-O bonds are created, leading to the formation of an insoluble film that stops the development. To prevent the formation of this film, several studies suggest adding NaCl to weaken the Si-O bond [26]. Therefore, the development solution consists of MFCD-26 diluted in water at 1:1 ratio, and 4% in weight of NaCl added as a catalyst and contrast enhancer [27]. The solution is kept at 293 K during development. Dilution makes it possible to make the development reaction more gradual and, therefore, more homogeneous at the cost of an extension of the development time. The latter will be discussed in more detail below.

In the third step, according to sub-process 3 (Figure 2), the cavity is covered with a stack of organic layers evaporated through a first hollow mask (Mask5). The process is finalized by vacuum evaporation of the aluminum cathode through a second hollow mask (Mask 6). Both evaporations are performed without exposure to ambient air so as to prevent moisture and oxygen degrading the organic semiconductor properties. Therefore, the change of Mask 5 to 6 and the alignment of Mask 6 (Figure 2) are carried out in a glove box (1 ppm N_2_) connected to the vacuum evaporator. A challenge is, therefore, to perform the alignment of Mask 6 with the cavity in the glovebox, which prevents the use of mask aligner and, therefore, requires operation with bare hands and via the naked eye. Similarly, no photolithography step can be performed after the evaporation tasks because organic semiconductors would be dissolved in solvents. This is a severe constraint in the fabrication, which explains part of the difficulty in cavity integration. The precision of the alignment better than 50 µm is related to the width of the smallest dimension of Mask 6 (100 µm) to be positioned at the center of a DFB photonic nanostructure measuring 200 × 200 µm^2^ (Figure 1b). Marker slits on the hollow mask and marker lines on the substrate with Vernier effect are used to accurately perform naked-eye sub-100 µm alignment [16].

### 2.3. Process Optimization

As explained previously, we primarily investigated the ITO thickness, the dose, and the time of development to determine the appropriate fabrication parameters and their impact on the grating geometry. It is crucial to carefully optimize the process parameters in order to achieve the required accuracy.

The key objective is to ensure that no residual resist layer remains in the non-exposed areas while avoiding over-development, which can result in a reduction in the grating duty cycles. In the first scenario, leaving a residual resist layer may lead to charge carrier injection issues, causing a significant increase in device resistivity, uneven injection, and localized heating. In extreme cases, it could even prevent proper injection altogether. In the second scenario, over-development will undoubtedly degrade the cavity efficiency, specifically the Q-factor by modifying $\Lambda_{L}$ the line width and the grating efficiency.

- ITO thinning Process

**Figure 3:**
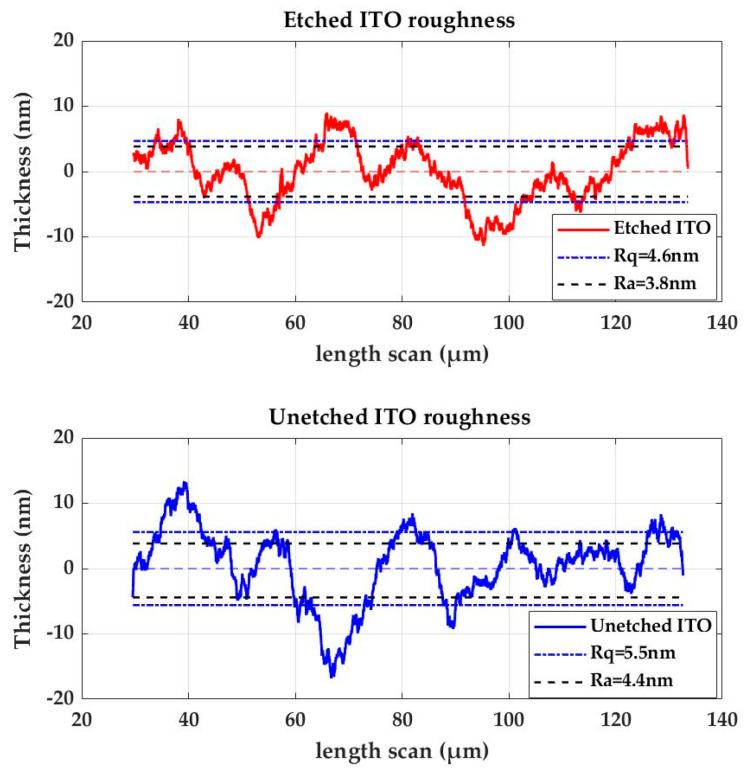
Roughness of the etched ITO in the active area (orange line) and that of the unetched ITO part (blue line).

The optimal ITO thickness is the result of a trade-off between two aspects: an optical aspect and an electrical one. From the optical perspective, the optimal confinement factor maximizing the optical field in the organic layers is achieved with 50 nm thin ITO, as explained in [16]. However, from the electrical point of view, increasing the thickness of the ITO layer helps to decrease the serial resistance to improve the carrier injection and to prevent voltage drop across the OLED and to minimize localized heating. The optimal standard commercial ITO thicknesses for OLEDs are conventionally 140 nm (15 Ω/☐) and 340 nm (5 Ω/☐). To achieve 50 nm thin ITO active area while keeping the rest of the ITO arm as thick as possible, standard commercial ITOs are etched by inductively coupled plasma (ICP), as shown in Figure 2. A CH4/Cl2-based plasma etching process using parameters shown in Table 1 was applied to etch the 100 µm × 200 µm region centered in the middle of the anode arm (Figure 1b). This etching method offers the advantage of favoring a vertical engraving profile by combining both a chemical etching effect (plasma) and a highly anisotropic physical etching effect (ionic bombardment). However, it lacks selectivity. Further investigation led to the identification of the optimal ITO/photoresist etching selectivity using He/Ar [28,29]. Figure 3 shows the roughness of the etched ITO in the active area and that of the unetched ITO part, which is *Rq* = 4.6 nm (*Ra* = 3.8 nm) and *Rq* = 5.5 nm (*Ra* = 4.4 nm), respectively. This small difference in roughness is compatible with an OLED deposition of hundreds of nm.

- Dose study

We conducted experiments using three different samples with varying ITO layer thicknesses (50 nm, 140 nm, 340 nm). The difference in thickness between the transparent and conductive layers results in variations in the distribution of electron charge during exposure. As a consequence, different optimal doses are required to achieve the same pattern. In this study, the digital mask consisted of a DFB micro-cavity pattern with a line width of 114 nm and an interline spacing of 97 nm.

**Figure 4:**
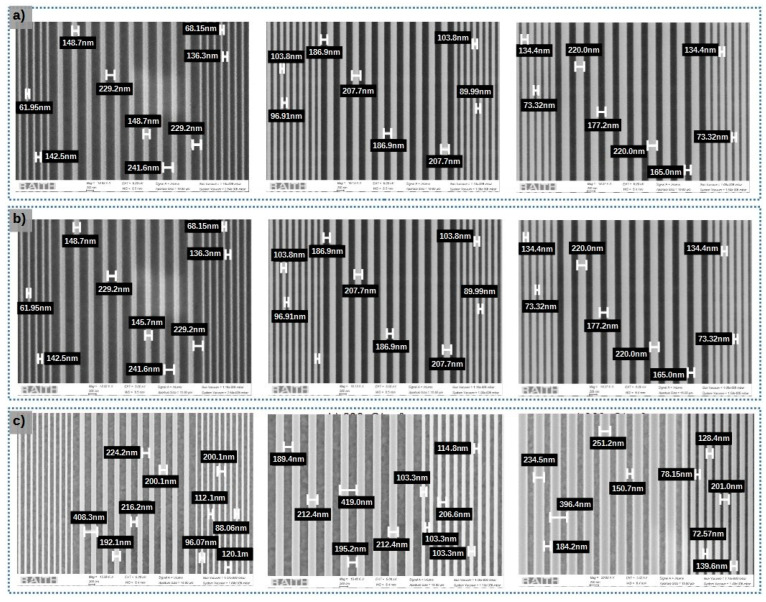
SEM image for dose test for samples with; (**a**): 340 nm ITO thick for 400 µC/cm^2^, 600 µC/cm^2^ and 800 µC/cm^2^, (**b**) 140 nm ITO thick for 400 µC/cm^2^, 600 µC/cm^2^ and 800 µC/cm^2^, (**c**): 50 nm ITO thick for 500 µC/cm^2^, 600 µC/cm^2^ and 700 µC/cm^2^.

**Figure 5:**
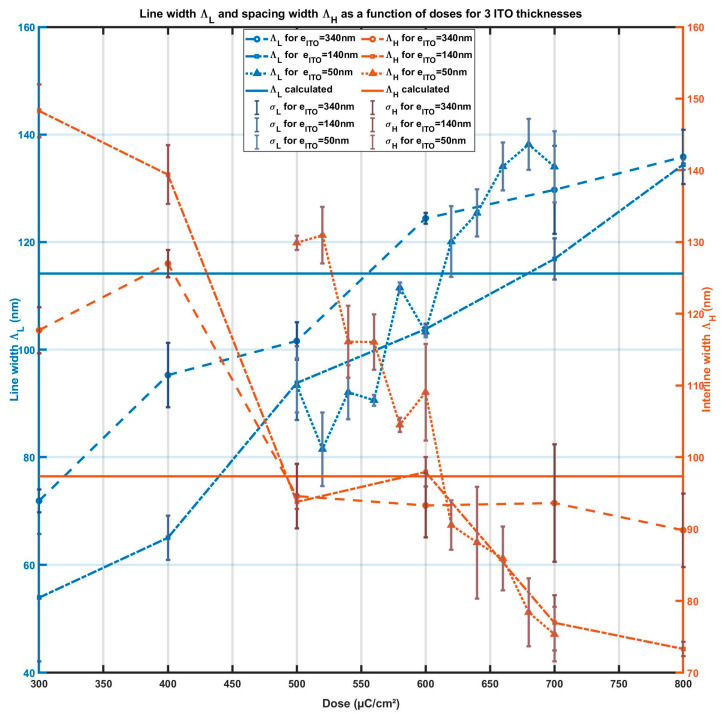
Line width $\Lambda_{L}$ and interline spacing $\Lambda_{H}$ as a function of the e-beam dose of exposure for 3 ITO thicknesses: Blue curves present $\Lambda_{L}$ the width of the SiO_2_ lines. Orange curves correspond to the spacing width $\Lambda_{H}$; dashed lines correspond to 340 nm thick of ITO coating, dash-dotted lines for 140 nm thick of ITO coating, dotted lines correspond to 50 nm thick of ITO coating. Solid lines are the targeted value of $\Lambda_{L}$ (blue) and of $\Lambda_{H}$ (orange).

To determine the optimal doses, we varied the doses within a range of 300 µC/cm^2^ to 800 µC/cm^2^. Figure 4 presents the SEM observations of the electron-beam lithography for ITO thicknesses of 340 nm for line a, and ITO thicknesses of 140 nm for line b, with doses of 400 µC/cm^2^, 600 µC/cm^2^, and 800 µC/cm^2^. Line c presents the SEM observation of e-beam lithography for ITO thicknesses of 50 nm and doses of 500 µC/cm^2^, 600 µC/cm^2^, and 700 µC/cm^2^. Figure 5 presents the measured line width $\Lambda_{L}$ (orange curves) and interline spacing width $\Lambda_{H}$ (blue curves) as a function of the doses and for ITO thicknesses of 340 nm (solid line), 140 nm (discontinued lines), and 50 nm (dash lines). The targeted values are presented by a horizontal magenta line for $\Lambda_{L}$ the SiO_2_ line width and cyan line for $\Lambda_{H}$ the interline spacing. This study confirms that the optimal dose depends on the thickness of the ITO layer. Our analysis leads us to the following conclusion: the optimal charges per area for the samples with ITO thicknesses of 340 nm, 140 nm, and 50 nm are 500 µC/cm^2^, 600 µC/cm^2^, and 620 µC/cm^2^, respectively.

- Time development study

**Figure 6:**
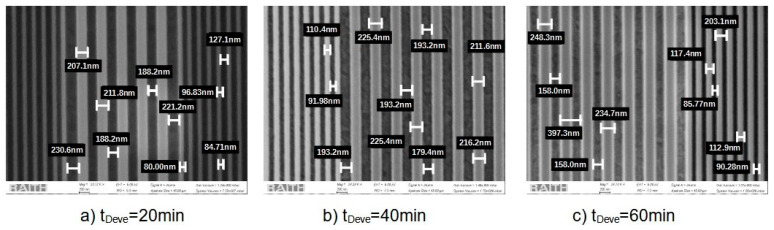
SEM image for development time test for samples with 140 nm ITO thick.

**Figure 7:**
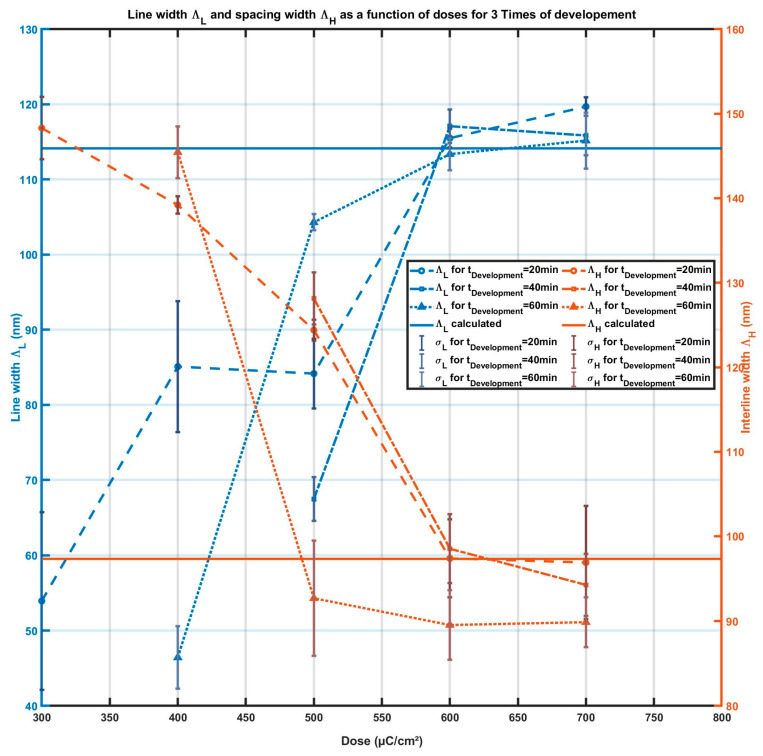
Line width *Λ_L_* and spacing *Λ_H_* as a function of the dose of exposure for 3 development times: Blue curves correspond to the width of the SiO_2_ lines *Λ_L_*. Orange curves corresponds to the spacing width *Λ_H_*; dashed lines for 20 min, dash-dotted lines correspond to 40 min, dashed lines correspond to 60 min. Solid lines are the targeted values of *Λ_L_* (blue) and *Λ_H_* (orange).

The time of development also needs to be adjusted according to the previous parameters. For this study, which uses a 140 nm thick ITO anode, the targeted line width and interline spacing are $\Lambda_{L}=114\;\mathrm{nm}$ and $\Lambda_{H}=97\;\mathrm{nm}$. For several doses from 300 µC/cm^2^ to 800 µC/cm^2^, we study the impact of the development time on the pattern accuracy. Figure 6 presents the SEM observations of the electron-beam lithography for different development times (20 min, 40 min, and 60 min) for ITO thicknesses of 140. Figure 7 shows $\Lambda_{L}$ the measured line width and $\Lambda_{H}$ the interline spacing as a function of the dose for different development times: 20 min (solid lines), 40 min (discontinued lines), and 60 min (dashed lines). The targeted values are represented by the cyan line for interline spacing and the magenta line for the SiO_2_ line. The optimal development time is between 20 and 40 min.

The optimal parameters are reported in Table 2. Note that the relevant dose depends on the ITO thickness.

With these parameters and for a development time of 20 min, the line width and the interline spacing are found to be 116 ± 2 nm and 97 ± 2 nm, which are 3.5% and 1.7% relative errors. For development time of 40 min, the relative errors are 4.3% and 3%.

## 3. Results

**Figure 8:**
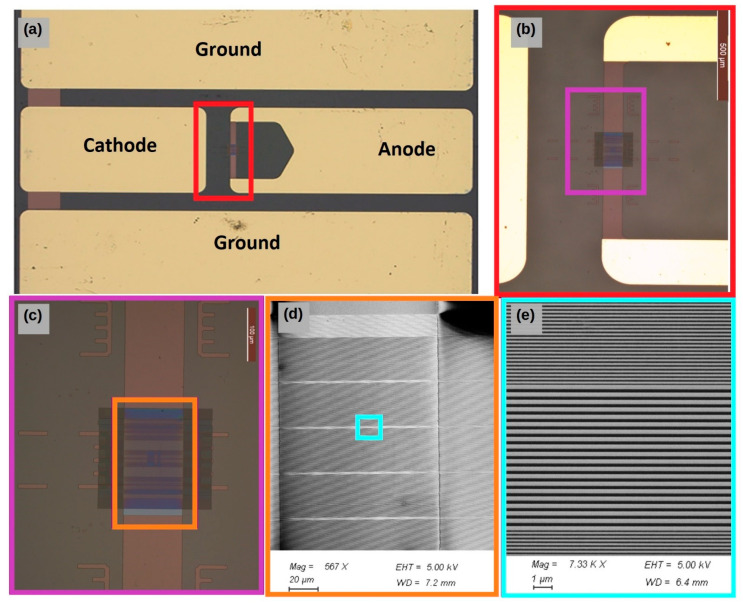
Top views of device: optical microscope: (**a**) the CPW electrodes, (**b**) zoom of (**a**) to present the position of the cavity relative to the electrodes, (**c**) zoom of (**b**) showing the mixed-order cavity in the thinned ITO anode, (**d**) SEM observation of the mixed-order cavity and (**e**) zoom of (**d**) showing the first-order gratings separated by the second-order grating.

Figure 8 shows pictures of the well-aligned cavity on the ITO anode and process, with the parameters in Table 2.

We fabricated CPW electrodes by etching and gold-metalizing a 140 nm (or 340 nm) thick ITO layer. The CPW electrodes are made with 1500 µm large central lines separated from ground planes by gaps, as shown in Figure 8a.

Intermediate ends of the CPW electrodes are terminated with un-metallized 100 µm large and 800 µm long ITO arms (vertical in the picture), in the middle of which a 250 µm long region was thinned down to 50 nm to improve the confinement factor and to maximize the index contrast. This region is intended for the µ-cavities to be integrated, as illustrated in Figure 8b. It appears as a pale-bluish rectangle, whereas the un-thinned ITO arm is brownish-rose. The implemented µ-cavities appear as a dark square overlapping the thinned sections, as shown in Figure 8c. Note the presence of markers and rulers to facilitate the alignments and the localization and centering of the DFB pattern with respect to the ITO arm.

The 200 µm × 200 µm µ-cavity pattern fabricated by e-beam lithography in hydrogen silsesquioxane (HSQ) negative-tone e-beam resist consists of a first-order grating interrupted five times with five second-order gratings. It starts and ends with second-order gratings. The reason why five cavities are fabricated is to make sure that at least one of them is covered by the aluminum cathode in the case the latter is not perfectly centered. A large scanning electron microscope (Raith Pioneer) observation presents several second-order gratings (light-grey lines) interrupting the first-order gratings (dark-grey line), as shown in Figure 8d. For a cavity resonance at *λ* = 622 nm, the 300 nm thick HSQ first-order gratings are patterned with $\Lambda_{L1}=107\;\mathrm{nm}$ wide quarter-wavelength lines spaced from each other with $\Lambda_{H1}=91\;\mathrm{nm}$ wide quarter-wavelength interline spacing, while second-order gratings show $\Lambda_{L2}=214\;\mathrm{nm}$ and $\Lambda_{H2}=182\;\mathrm{nm}$. Figure 8e shows the quarter-wavelength mirrors interrupted with 19 half-wavelength defects pairs plus 1 made of second-order lines and interline spacing observed with a scanning electron microscope.

**Figure 9:**
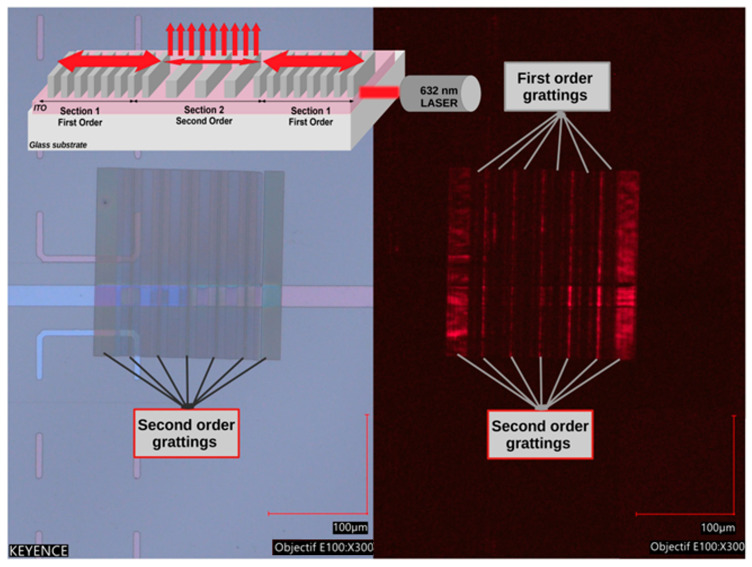
Optical response of the cavity under grazing light injection.

The light is intended to be extracted from the cavity through a diffractive mode of the second-order Bragg grating, perpendicular to the direction of propagation. To experimentally illustrate the proper functioning of the cavity, we inject light from a laser-collimated beam in the cavity plane (top of Figure 9 up). A 1 mW diode laser emitting a 632 nm wavelength close to the resonance wavelength is used.

A top view of the sample under ambient illumination and without laser injection is shown in Figure 9 left, where the ITO horizontal stripe exhibits two colors; on the left part, the 50 nm thick stripe is light-purple-blue, whereas on the right part, the 140 nm thick ITO stripe is dark-rose. The 200 µm × 200 µm DFB pattern overlaps the horizontal stripe and, on each ends, the second-order sections appears slightly darker as well as the narrow second-order sections. Figure 9 right, shows the same pattern in the dark with 632 nm laser light injected from the right side. The light intensity decreases from right to left, with larger illumination corresponding to second-order grating sections. The second-order sections located on both the 50 nm thin and 140 nm thick ITO sections clearly diffract light contrary to first-order sections. The ITO horizontal stripe is visible on the right side, where the thickness is 140 nm, which allows several waveguided modes to exist contrary to the 50 nm thin ITO stripe part. This confirms that second-order grating effectively couples light out of the plan of the grating.

**Figure 10:**
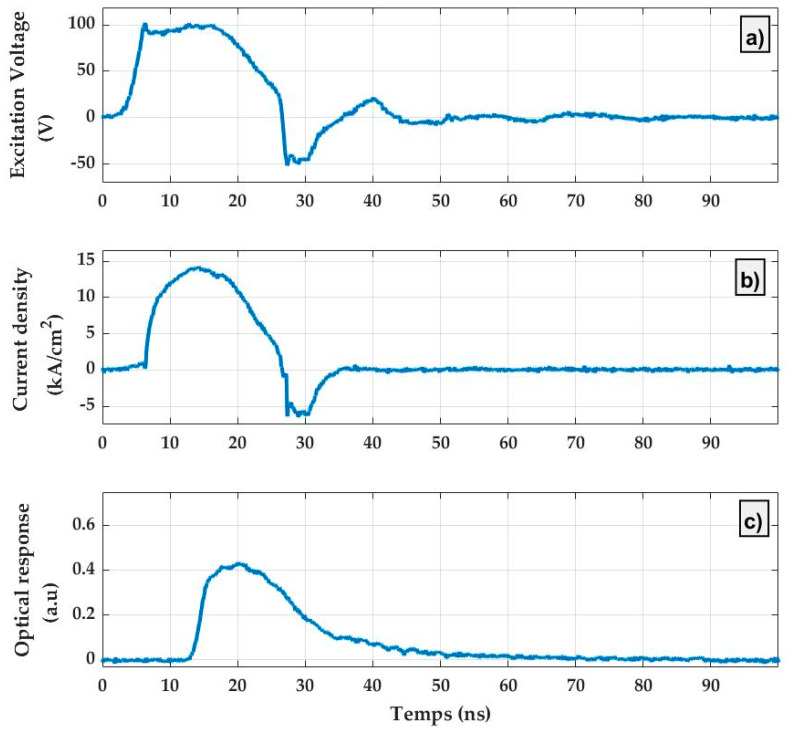
Time-resolved response of an OLED with a mixed-order DFB micro-cavity subjected to a single pulse of 105 V and of 20 ns in duration: (**a**) excitation signal, (**b**) current density response, and (**c**) optical response.

Figure 10 shows the time responses of the current density and the light output in the arbitrary unit of an OLED with a mixed-order DFB micro-cavity subjected to a single pulse of 105 V and 20 ns in duration. A current density as high as 14 kA/cm^2^ is measured, which is among the highest current density measured with an OLED so far [30,31]. The measured light output corresponds to 1.7 µW. This is an indication that the device is electrically operational, including in the nanosecond pulse regime, despite the lower uniformity of the thinned ITO and the presence of the grating in the active area.

## 4. Conclusions

In the context of the quest for the Organic Laser Diode, we reported the multiscale fabrication process optimization of mixed-order distributed-feedback micro-cavities integrated in nanosecond-short electrical pulse-ready organic light-emitting diodes. The targeted device integrates three elements with different dimensional and precision requirements. Firstly, a 100 × 200 µm^2^ multisection DFB micro-cavity made of 198 nm and 396 nm first- and second-order grating periods was proposed. The precision on the grating periods needed to tune the cavity resonance wavelength precisely to the electroluminescence peak of the organic laser gain medium is 5 nm. The second elements are microwave electrodes, allowing nanosecond and sub-nanosecond electrical pulses to be delivered efficiently to the OLED, so as to achieve intense excitation at the levels of the laser threshold. The third element is the organic multilayer stack with a 100 µm width aluminum cathode. The latter is centered on the 100 × 200 µm^2^ DFB micro-cavity. Because organic semiconductors suffer from oxygen and moisture, the cathode is vacuum deposited through a 100 µm width hollow mask previously hand-aligned in a 1 ppm-N_2_ glovebox without exposure of the samples to air. For the same reasons, post-evaporation photolithographic steps are forbidden.

Additionally, fabrication uncertainties cause deviation in the resonance wavelength with respect to the peak wavelength of the gain medium electroluminescence.

The whole elaborated process consists of 20 steps gathered in three sub-processes. Above the previously patterned CPW electrodes, the DFB micro-cavities were patterned by e-beam lithography before the OLED stack was vacuum deposited. The e-beam patterning of a negative-tone hydrogen silsesquioxane (HSQ) resist was finely optimized to reduce the fabrication uncertainties affecting the DFB micro-cavity period. Two studies were conducted to identify the optimal parameters affecting the line widths and the interline spacing and, thus, the period of the mixed-order grating. First, the optimal dose for exposure of the 300 nm thick negative-tone e-beam HSQ resist was studied for different substrates covered by different thicknesses of ITO in order to take into account the difference in charge flow. It was found that the optimal doses for ITO thicknesses of 340 nm, 140 nm, and 50 nm are different and are 500 µC/cm^2^, 600 µC/cm^2^, and 620 µC/cm^2^, respectively. Second, different development times were studied. The optimal development time was found to be 20 min for a 1:1 MFCD26/H_2_O solution containing 4% of NaCl, resulting in a relative error of 3.5% in the grating period. With the selected parameters, the targeted 5% relative error on the grating resolution is achieved, allowing for optimal tuning of micro-cavity resonance to the organic semiconductor electroluminescence spectrum.

Finally, we provide overviews of the micro-cavity and of the CPW electrodes, showing the different scales at stake in the fabrications and in the alignments. When subjected to side illumination from a 632 nm laser, the second-order gratings clearly diffract light contrary to first-order gratings, indicating proper functioning. Moreover, it is shown that the integration of a micro-cavity in the OLED does not hinder the electrical operability of the device, even under intense excitation in the nanosecond pulse regime. A current density as high as 14 kA/cm^2^ was measured. As a perspective, we plan to identify and use organic semiconductors with higher effective laser gain in the organic heterostructure of the OLED to potentially reach the laser threshold.

## Figures and Tables

**Table 1:** ITO etching recipe.

Pressure [mTorr]	T [°C]	RF Power [W]	LF Power [W]	Cl_2_ Flow [sccm]	C_2_H_4_ Flow [sccm]	Ar Flow [sccm]	He Flow [sccm]
10	20	200	300	30	7	50	10

**Table 2:** E-beam optimal parameters.

Area write field	200 µm × 200 µm
Area step	0.02 µm
Acceleration voltage	20 kV
Diaphragm diameter	15 µm
Working distance	6.5 mm
Pitch current	Between 60 and 80 pA
Dose	620 µC/cm^2^ for 50 nm of ITO 600 µC/cm^2^ for 140 nm of ITO 500 µC/cm^2^ for 340 nm of ITO
Developer	MFCD 26: H_2_O + NaCl (1:1, 4% of NaCl)
Time development	Between 20 and 40 min

## Data Availability

Data are contained within the article.
